# Fatigue in children who have recently completed treatment for acute lymphoblastic leukemia: a longitudinal study

**DOI:** 10.1186/s12955-024-02241-2

**Published:** 2024-03-22

**Authors:** S. Walsh, M. Mulraney, M.C. McCarthy, Cinzia R. De Luca

**Affiliations:** 1 Institute for Social Neuroscience, ISN Psychology, Heidelberg, VIC Australia; 2 Clinical Sciences, Murdoch Children’s Research Institute, Melbourne, VIC Australia; 3 Department of Paediatrics, University of Melbourne, Melbourne, VIC Australia; 4 Children’s Cancer Centre, Royal Children’s Hospital, 50 Flemington Road, Parkville, VIC 3052 Australia

**Keywords:** ALL, Leukemia, Fatigue, Chemotherapy, Pediatric, Oncology

## Abstract

**Background:**

This study examined fatigue in patients treated for childhood acute lymphoblastic leukemia (ALL) over a 2-year period (3- to 27-months post-treatment completion), from the perspective of children and parent caregivers, compared to a healthy comparison group.

**Methods:**

Eighty-three patients (4–16 years at enrolment) and their parents, reported on the child’s fatigue using the Pediatric Quality of Life Inventory– Multidimensional Fatigue Scale (PedsQL-MFS), at 3- 15- and 27-months post-treatment completion, and 53 healthy children and their parents reported on fatigue across the same timepoints.

**Results:**

Parent proxy-reporting showed that parents of ALL patients reported more total fatigue than parents of the comparison group at all time points, with all subscales elevated (general, cognitive, and sleep/rest fatigue). In contrast, patient self-report of fatigue over this period differed from the comparison children for the general fatigue subscale only. Self-reported total fatigue was worse than the comparison group at the 27-month timepoint, with cognitive and sleep/rest fatigue symptoms contributing to this difference. Expected improvements in fatigue over time were not evident in either patient or parent report and no demographic risk factors were identified. Parents and children from both groups reported significantly more fatigue at all time points compared to commonly utilised normative population data.

**Conclusions:**

Patients treated for childhood ALL are impacted by fatigue symptoms in the post-treatment and early survivorship period. These findings highlight that patients in the 2-years following treatment require increased symptom surveillance and may benefit particularly from interventions that target cognitive and sleep/rest fatigue.

**Supplementary Information:**

The online version contains supplementary material available at 10.1186/s12955-024-02241-2.

## Background

Acute lymphoblastic leukemia (ALL) is the most common childhood malignancy, and most affected children (> 90%) will become long-term survivors [1, 2]. With improved survivorship, a focus on quality-of-life outcomes, including the late effects of treatment, has become increasingly important. Survivors and caregivers have identified fatigue as one of the most prevalent and distressing late effects experienced following childhood cancer treatment [3, 4]. Fatigue refers to a physical, emotional, and/or cognitive exhaustion that is not proportional to recent activity [5]. Fatigue can negatively impact the lives of pediatric cancer survivors, increasing levels of depression, diminishing neurocognitive and behavioral functioning, and impacting academic achievement and overall quality of life [6–8].

Recent research has identified increased fatigue levels in children with ALL (and other leukemias) on active treatment [9, 10]. However, findings have been mixed with respect to the rate and impact of fatigue symptoms following treatment completion [7, 11–13]. Studies assessing the early survivorship phase directly following treatment, provide some evidence that fatigue symptoms may improve over time [14, 15]. Yet, elevated fatigue has been reported in approximately half of all long-term survivors [16], with chronic deficits almost three times more prevalent than the healthy population in survivors up to 20 years post-diagnosis [17]. Conversely, other studies identified no differences in reported fatigue of survivors during early and late survivorship phases compared to healthy population norms and controls [12, 18]. Findings are also varied with regard to risk factors associated with health-related quality of life in childhood cancer survivors, with only some studies finding sex, age, and treatment-related factors to be associated with fatigue in this population of survivors [8, 19].

Variability in findings may be partly attributed to limitations in study design, including variations in diagnosis, time since treatment completion, treatment regimens and a lack of adequate comparison groups, which make it difficult to draw definitive conclusions regarding the prevalence and impact of fatigue [8]. There has been a reliance on cross-sectional and longer-term survivorship studies (> 5 years) [7, 12], or those including treatment regimens that no longer reflect modern protocols [20, 21], which has hampered development of appropriate early interventions. Longitudinal data, mapping fatigue symptoms in the early post-treatment period, is needed to inform the development of timely interventions.

Additionally, although child and caregiver reports of fatigue appear to be correlated, some degree of variation between informants has been seen [11, 22, 23]. These differences have been attributed to children providing a more subjective representation of fatigue while parent reporting may reflect different perspectives, including causative and environmental factors and differences compared to siblings or peers [24]. Given conflicting findings regarding the presence and course of fatigue symptoms in patients treated for ALL, research using multiple informants to examine the clinical trajectory of fatigue symptoms from treatment into survivorship is needed.

The current study aimed to examine fatigue from the perspective of child and adolescent patients treated for ALL (hereafter referred to as the patient group) and their parents, from 3-months to 27-months post-treatment completion, compared to a healthy comparison group. These data form part of a longitudinal study, the *ALLaboard study*, that aimed to examine the trajectory of child-related cognitive and behavioral outcomes as well as family wellbeing, for approximately two years post-ALL treatment. We hypothesized that both the patient group and their parents would report higher levels of fatigue than a comparison group of healthy peers, over the 2-year period. We also hypothesized that child and parent reports of fatigue would decrease over time for the patient group, but not the comparison group. Exploratory aims included (i) examining which domains of fatigue (general fatigue, cognitive fatigue, sleep/rest fatigue) are significantly impacted, (ii) whether there is an elevated risk for fatigue at a particular time-point following treatment completion, and (iii) whether sex, age at diagnosis, or treatment intensity are related to an increased risk of fatigue at 27-months post treatment completion.

## Method

### Participants

#### Patient group

Patient participants were children aged 4–16 years, who had completed chemotherapy-only treatment for ALL, no more than 3-months prior to enrolment, as well as a parent caregiver. Patients had completed treatment at the Royal Children’s Hospital (RCH) or Monash Children’s Hospital (MCH) in Melbourne, Australia, and were recruited prospectively between October 2013 and December 2017. Participants were ineligible for the patient group if they or their parent had insufficient English to complete the assessments, were born prematurely (< 30 weeks), had a pre-existing neurodevelopmental or neurological disorder, had received radiation treatment, had relapsed, were receiving further treatment, or were in palliative care. Patient participants were treated with intrathecal and intravenous methotrexate (MTX) according to the Children’s Oncology Group protocols (Standard risk: AALL0331, AALL0932; High risk: AALL0434, AALL0232, AALL1131).

#### Comparison group

Comparison group participants were a convenience sample of healthy children and a parent caregiver. They were recruited alongside the patient sample via patient participant families inviting a friend of the same age (< 1 year) and sex to participate, as well as a small number of children recruited to this group through advertising at the RCH. They were seen over the same data collection period, often in parallel with their ‘buddy’ patient participant. Participants were ineligible for the comparison group if they or their parent had insufficient English to complete the assessments, were born prematurely (< 30 weeks), had a pre-existing neurodevelopmental or neurological disorder, or a history of malignant disease.

### Procedure

Parents and patients were informed about the study by their treating oncologist at the child’s final on-treatment appointment. For participating families, the research team obtained written consent from parents and children ≥ 12 years of age, and verbal assent from younger children. Study assessments were conducted by a neuropsychologist, or trained research assistant with a minimum undergraduate training in psychology under the supervision of a clinical neuropsychologist. Patient and comparison participants attended the hospital to complete various assessments across five timepoints, with fatigue measures collected at timepoint 1 (3-months post-treatment), timepoint 3 (15-months post-treatment), and timepoint 5 (27-months post-treatment). As this paper examined fatigue outcomes only, these timepoints are hereafter referred to as Time 1, Time 2, and Time 3, respectively. Both child and parent caregiver participants reported on the children’s level of fatigue, with children using the age-appropriate form of the fatigue measure. The 19 participants who were under 5 years old (mean 4.6 years) at the first timepoint, completed the 5–7 year old form of the measure (Supplementary Table S1).

### Measures

#### Demographic information

Relevant demographic information was obtained via a parent completed questionnaire.

#### Intensity of treatment rating scale (ITR-3)[25

Information regarding diagnosis and treatment protocols were extracted from hospital medical records. Treatment intensity was classified using the Intensity of Treatment Rating Scale − 3.0 (ITR-3). This scale uses treatment modality and risk level to rate the intensity of pediatric cancer treatments as, 1 = *least intensive*, 2 = *moderately intensive*, 3 = *very intensive*, 4 = *most intensive*. The ITR-3 has demonstrated reliability and validity when assessing modern treatment protocols [25]. In the current study, a medical oncology consultant and the study’s principal investigator completed the measure, with an inter-rater reliability of 1.0. All treatments in the current study were rated as level 2 (standard risk = AALL0331, AALL0932) or 3 (high risk = AALL0434, AALL0232, AALL1131).

#### Pediatric quality of life inventory– multidimensional fatigue scale (PedsQL-MFS) [26

The Pediatric Quality of Life Inventory– Multidimensional Fatigue Scale (PedsQL-MFS) is an 18-item questionnaire designed to measure fatigue. There are parent proxy-report and child self-report forms available, with the current study utilising the 5–7 years and 8–18 years forms of the measure. The measure consists of three subscales, general fatigue (6 items), cognitive fatigue (6 items), and sleep/rest fatigue (6 items), and produces a total score which is a composite of all items. Participants indicate the extent to which the child has experienced difficulties in the past month on a scale from *never* (0) to *almost always* (4). Items are reverse scored, transformed onto a linear scale, summed, and divided by the total number of items. Final scores range from 0 to 100, with higher scores indicating less fatigue. The PedsQL-MFS has demonstrated strong psychometric properties [26], and these were upheld across the current study *(α*s = 0.75–0.88 5–7 years; *α*s = 0.90–0.91 8–18 years; *α*s = 0.94–0.95 parent).

#### Statistical analysis

Analyses were completed in Stata version 17.0. Descriptive statistics were calculated to examine the mean fatigue (SD) scores at each time point for patients and comparison children. For Hypothesis 1 (that the patient group would report greater fatigue than the comparison group), fatigue scores between the groups were compared using mixed effects linear regression models. This involved fitting a single mixed model to the Time 1, Time 2, and Time 3 data. Two models were fitted: one using parent-reported fatigue, and one using child-reported fatigue. The models included a random effect to account for the correlation between repeated measures within an individual, fitting a separate between- and within-cluster variance at each time point. For Hypothesis 2 (that fatigue would reduce over time in the patient group but not the comparison group), the interaction term between group and time was added to the mixed effects linear regression models. We plotted the mean fatigue scores for each group over time, including a reference line in the figures representing the norms for the PedsQL-MFS [26]. Single sample t-tests were used to compare the study fatigue scores to normative data (Supplementary Table S2). Finally, we conducted multivariate linear regressions to determine whether sex, age at diagnosis, or treatment intensity predicted fatigue scores in the patients at Time 3. Results are presented as mean differences, 95% confidence intervals (CIs), and *p* values. Cohen’s d effect sizes are also reported with effect sizes of ∼0.20 considered small, ∼0.50 moderate, and ∼0.80 as large [27].

## Results

### Sample characteristics

A total of 83 patients and parents, along with 53 healthy peers and their parents were enrolled over the course of the study (Table 1). Child participants were aged between 4 and 16 years at Time 1. Groups were well matched for sex and age with no statistically significant differences. There was some variation in sample size across the study timepoints due to attrition and relapse, participants onboarded at the second timepoint, and incomplete measures for some participants at each timepoint (see Supplementary Figure S3 & Supplementary Table S4). However, the groups did not differ on demographic factors at any timepoint.

**Table 1:** Sample characteristics

	Patient		Comparison	
N	83		53	
**Sex, n (%)**				
Male	47	(56.6)	25	(47.2)
Female	36	(43.4)	28	(52.8)
**Age**				
Mean (SD)	8.1	(3.2)	8.4	(3.5)
**Treatment intensity, n (%)**				
Standard	49	(59.0)	-	
High	34	(41.0)	-	
Parent relationship to child, n (%)*				
Mother	72	(87.8)	48	(92.3)
Father	10	(12.2)	4	(7.7)

* Unreported *n* = 1 for both groups

### Child self-reported fatigue scores

**Fig. 1:**
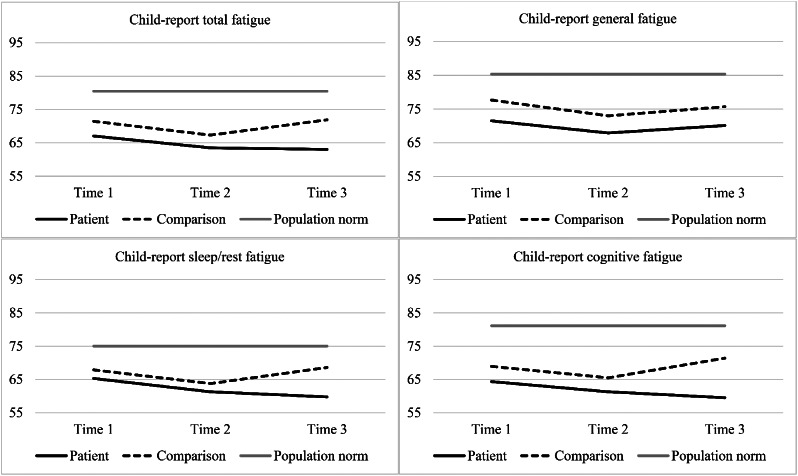
Mean child-reported fatigue scores over time for the patient group, comparison group, and normative data

Children’s fatigue scores at each time point can be seen in Table 2; Fig. 1. The results of the mixed effects regression models examining child-reported fatigue scores between the two groups over time can be found in Table 3. There was no main effect of group on the total fatigue scale. There was a main effect of group on the general fatigue subscale only, whereby the patient group tended to report more general fatigue than comparison children, with a small to moderate effect. The interaction between group and time (Table 3) was not significant for the total fatigue scale, nor any of the subscales, indicating that the groups did not significantly differ from each other in how their fatigue changed over time. Although the group main effect and group by time interactions were not significant, at Time 3, the mean scores differed significantly between the groups, such that the patient group reported significantly more fatigue on the total, sleep/rest, and cognitive fatigue subscales with moderate effect sizes. Supplementary analyses indicated that both the patient group and comparison group were significantly more fatigued than population normative data, on total fatigue and all subscales, and at every time point (Supplementary Table S2). Age at diagnosis, sex, and treatment intensity were not found to be significant risk factors for elevated fatigue at Time 3 (Table 4).

### Parent proxy-reported fatigue scores

**Fig. 2:**
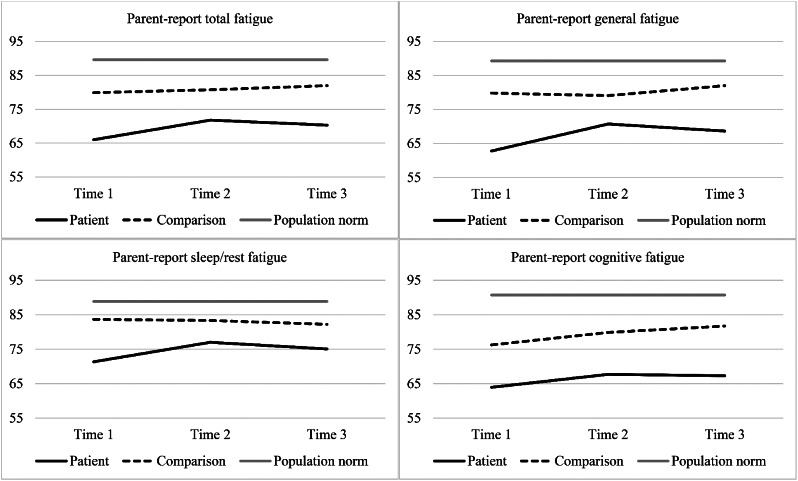
Mean parent-reported fatigue scores over time for the patient group, comparison group, and normative data

Parent reported fatigue scores are outlined in Table 2; Fig. 2. As shown in Table 3, there was a significant main effect of group on the total fatigue scale, whereby parents from the patient group reported significantly greater fatigue than the comparison group, with a moderate to large effect size. A main effect was also seen across all three subscales (effect sizes ranging from moderate to large). The mean differences show that parents rated the patient group as experiencing greater general, cognitive and sleep/rest fatigue than the comparison group at each time point. The group by time interaction for the total fatigue score was not significant (see Table 3). There was a significant group by time interaction for the general fatigue subscale only, indicating that the patient group rated their child’s general fatigue as reducing over time, while the comparison group did not differ in their ratings of general fatigue over time. Supplementary analyses found that both the patient group and the comparison group were significantly more fatigued than the normative data according to parents, on total fatigue and all subscales, at every time point (Supplementary Table S2). Age at diagnosis, sex, and treatment intensity were not found to be significant risk factors for elevated fatigue at Time 3 (Table 4).

**Table 2:** Fatigue scores by child and parent report

	Patient M (SD)			Comparison M (SD)		
Child report	*Time 1* *(n = 76)*	*Time 2* *(n = 73)*	*Time 3* *(n = 67)*	*Time 1* *(n = 52)*	*Time 2* *(n = 43)*	*Time 3* *(n = 43)*
Total fatigue	67.05 (19.01)	63.49 (17.06)	63.02 (20.03)	71.47 (14.91)	67.31 (13.71)	71.90 (13.57)
General fatigue	71.49 (22.52)	67.87 (20.07)	70.09 (20.30)	77.64 (16.59)	72.97 (12.71)	75.68 (13.70)
Sleep/rest fatigue	65.30 (20.20)	61.30 (20.30)	59.76 (22.78)	67.87 (16.92)	63.76 (19.87)	68.60 (17.89)
Cognitive fatigue	64.36 (25.78)	61.30 (22.75)	59.51 (27.47)	68.91 (21.79)	65.50 (20.56)	71.41 (17.92)
Parent report	*Time 1* *(n = 76)*	*Time 2* *(n = 73)*	*Time 3* *(n = 68)*	*Time 1* *(n = 51)*	*Time 2* *(n = 42)*	*Time 3* *(n = 41)*
Total fatigue	66.01 (17.69)	71.80 (14.77)	70.32 (16.57)	79.90 (10.66)	80.75 (13.26)	81.98 (11.74)
General fatigue	62.77 (19.14)	70.72 (16.21)	68.63 (18.23)	79.82 (12.89)	79.07 (14.74)	82.01 (11.95)
Sleep/rest fatigue	71.33 (17.56)	77.00 (16.88)	75.06 (18.16)	83.66 (12.10)	83.33 (12.21)	82.22 (13.14)
Cognitive fatigue	63.93 (23.04)	67.69 (19.08)	67.28 (21.39)	76.23 (14.15)	79.86 (17.77)	81.71 (15.64)

Time 1 = 3-months post-treatment completion. Time 2 = 15-months post-treatment completion. Time 3 = 27-months post-treatment completion

**Table 3:** Comparison of child-reported and parent-reported fatigue scores between patients and comparisons over time

	Group Main Effect			Group by Time interaction		Time 1			Time 2			Time 3		
Child report	Mean difference^a^ (95% CI)	Effect size	*p*	Χ^2^ (df)	*p*	Mean difference^a^ (95% CI)	Effect size	*p*	Mean difference^a^ (95% CI)	Effect size	*p*	Mean difference^a^ (95% CI)	Effect size	*p*
Total fatigue	4.92 (-0.02, 9.86)	0.28	0.051	2.04 (2)	0.360	3.89 (-2.11, 9.90)	0.23	0.203	3.13 (-3.20, 9.46)	0.18	0.332	7.70 (1.30, 14.11)	0.45	**0.018**
General fatigue	5.34 (0.014, 10.53)	0.28	**0.044**	0.53 (2)	0.976	5.65 (-0.95, 12.25)	0.30	0.093	5.00 (-1.98, 11.99)	0.26	0.160	4.82 (-2.27, 11.90)	0.25	0.183
Sleep/rest fatigue	4.04 (-1.31, 9.39)	0.20	0.139	2.60 (2)	0.272	2.03 (-5.00, 9.07)	0.10	0.571	1.97 (-5.50, 9.44)	0.10	0.605	8.32 (0.74, 15.90)	0.41	**0.031**
Cognitive fatigue	5.78 (-0.81, 12.37)	0.24	0.086	2.33 (2)	0.311	4.26 (-4.02, 12.53)	0.18	0.313	3.10 (-5.66, 11.86)	0.13	0.488	10.21 (1.34, 19.08)	0.43	**0.024**
Parent report	Mean difference^a^ (95% CI)	Effect size	*p*	Χ^2^ (df)	*p*	Mean difference^a^ (95% CI)	Effect size	*p*	Mean difference^a^ (95% CI)	Effect size	*p*	Mean difference^a^ (95% CI)	Effect size	*p*
Total fatigue	12.10 (7.37, 16.82)	0.76	**< 0.001**	4.13 (2)	0.127	13.87 (8.63, 19.11)	0.87	**< 0.001**	9.47 (4.02, 14.91)	0.60	**0.001**	13.07 (7.57, 18.57)	0.82	**< 0.001**
General fatigue	13.41 (8.47, 18.34)	0.76	**< 0.001**	8.55 (2)	**0.014**	17.02 (11.26, 22.77)	0.97	**< 0.001**	8.56 (2.52, 14.60)	0.49	**0.005**	14.35 (8.23, 20.47)	0.82	**< 0.001**
Sleep/rest fatigue	9.49 (4.65, 14.33)	0.58	**< 0.001**	4.27 (2)	0.118	12.43 (6.85, 18.00)	0.76	**< 0.001**	6.80 (0.97, 12.64)	0.42	**0.022**	8.74 (2.83, 14.64)	0.53	**0.004**
Cognitive fatigue	13.22 (7.08, 19.36)	0.65	**< 0.001**	1.58 (2)	0.454	12.19 (5.37, 19.01)	0.60	**< 0.001**	12.87 (5.78, 20.00)	0.63	**< 0.001**	15.78 (8.62, 22.94)	0.78	**< 0.001**

^a^ Comparison group–patient group. Time 1 = 3-months post-treatment completion. Time 2 = 15-months post-treatment completion. Time 3 = 27-months post-treatment completion

**Table 4:** Associations between sex, age, treatment intensity and fatigue at Time 3 in the patient group

	Sex Β (95% CI)	Age at diagnosis Β (95% CI)	Treatment intensity Β (95% CI)
Child report			
Total fatigue	2.26 (-8.13, 12.65)	0.05 (-1.74, 1.83)	-5.40 (-17.55, 6.76)
General fatigue	5.15 (-5.31, 15.60)	0.29 (-1.51, 2.08)	-1.14 (-13.37, 11.09)
Sleep/rest fatigue	-1.82 (-13.21, 9.57)	-0.32 (-2.28, 1.64)	-9.10 (-22.42, 4.22)
Cognitive fatigue	2.97 (-11.26, 17.20)	0.10 (-2.34, 2.55)	-6.15 (-22.79, 10.49)
**Parent report**			
Total fatigue	-0.71 (-9.47, 8.06)	-0.99 (-2.50, 0.53)	-2.66 (-12.77, 7.44)
General fatigue	-3.77 (-13.29, 5.75)	-1.03 (-2.68, 0.61)	-0.47 (-11.44, 10.51)
Sleep/rest fatigue	-4.06 (-13.38, 5.25)	-1.60 (-3.21, 0.01)	-2.17 (-12.91, 8.57)
Cognitive fatigue	5.72 (-5.65, 17.09)	-0.32 (-2.29, 1.64)	-5.36 (-18.47, 7.75)

## Discussion

This longitudinal study explored fatigue over a 2-year period (3- to 27-months post-treatment completion), in patients treated for childhood ALL with modern chemotherapy-only treatment regimens. As anticipated, parents reported higher levels of total fatigue in the patient group than the comparison group over this period. In fact, parents rated patients as experiencing greater total, general, sleep/rest and cognitive fatigue, at every time point, to a meaningful degree given the moderate to large effect sizes. Fatigue levels were significant compared to both the healthy comparison children and the published normative data [26], signifying that parents of children treated for ALL find multiple aspects of fatigue to be impacting their child’s daily functioning. This is in line with earlier findings in which parents reported elevated fatigue in this population at an average of 36-months post-treatment [11]. Parent reports of fatigue for the patient group varied in the expected direction, indicating some reduction over time (i.e., PedsQL-MFS scores increased), however this change was only significant for the general fatigue subscale, indicating that while general fatigue symptoms seem to improve over time, cognitive and sleep/rest fatigue problems persist.

While the patient group did not self-report more total fatigue across this period, they were experiencing elevated symptoms of general fatigue compared to healthy children, and moreover, were significantly fatigued on all scales when compared to normative data [26]. Self-reports of patient fatigue moved in the opposite direction than expected, potentially indicating a slight worsening of symptoms. While this variation was not significant between the groups, it resulted in a discrepancy between survivor and peer reports so that by 27-months post-treatment, patients were reporting more symptoms of sleep/rest, cognitive, and total fatigue than their peers. Several factors may have contributed to this increase in reported fatigue symptoms, including the older age of respondents at this timepoint who may have more insight into their symptoms, an evolution of treatment-related cognitive challenges over time, and/or an increase in cognitive demand in both educational and daily functioning which resulted in a greater awareness of symptoms for the young person.

Despite previous indications that for survivors of childhood ALL, fatigue decreases during early survivorship and returns to levels consistent with the healthy population from 2 to 7 years post-treatment completion [18], the results of this study suggest that fatigue symptoms persist 27-months post treatment, with survivors impacted according to both the child and their parents. These findings are of concern given that patients are relatively unsupported once treatment has finished, with reduced access to healthcare services [28], but generally not yet eligible for longer term survivorship programs. There are currently missed opportunities to provide screening for fatigue and access to targeted interventions, a necessity echoed in a report on the surveillance of fatigue in childhood cancer survivors [29]. Providing this type of support may mitigate fatigue and attenuate the depressive symptoms and reduced quality of life which have been associated with fatigue in the childhood cancer population [12, 30].

This study did not identify any clear risk factors for ongoing fatigue in this patient population at 27-months post treatment. While these findings are in keeping with some earlier evidence indicating no age, sex, or treatment related risk factors to be present 12-months post treatment [31], alternate studies have identified age at diagnosis and female sex to be relevant to fatigue in the childhood cancer survivorship population [13, 32, 33]. As the current study examined the period directly following treatment completion, as opposed to patients further post-treatment, it seems possible that demographic risk factors go on to emerge further into survivorship.

Although no formal statistical comparisons were conducted, children often reported higher levels of fatigue comparative to their parents in both the patient and comparison groups in this study. Informant variance on fatigue between parents and children is often seen [11, 22, 34], and while the direction of this relationship has been mixed, parents reporting less fatigue comparative to children is a phenomenon seen in some studies utilising the PedsQL-MFS [34, 35]. This is a dynamic to be mindful of in a survivorship population, given that healthcare utilisation is often driven by the parent’s interpretation of symptoms and needs [36]. This provides further evidence for the necessity of utilising both parent and child informants, which assists in gaining richer and more accurate data on symptom burden [37].

Importantly, at all timepoints, parents and children from both the patient and comparison groups reported more fatigue than the commonly utilised normative values for the PedsQL-MFS [26]. The lifestyles of modern children are evolving, with the most apparent change being a rapid uptake of technology. Given that increased screentime has been associated with poorer sleep [38], and poor sleep outcomes are related to fatigue [39], this may be one contributing factor to a possible shift in current normative values. This highlights the importance of using contemporaneous control data, such as the age and sex-matched comparison group utilised in the current study. Caution should be taken in research when utilising previous normative values to determine whether the modern survivorship population are significantly fatigued.

Variability was also seen between patients treated for ALL and their parents on which domains of fatigue were considered most problematic. Parents provided the lowest scores (i.e., most fatigue) on cognitive and general fatigue, whereas children provided the lowest scores on cognitive and sleep/rest fatigue. A pattern of parents not attributing as many deficits within sleep/rest fatigue as children do themselves has been seen previously [22, 26]. While this may be representative of conceptual differences in fatigue between informants, experiences of insomnia, sleep disturbance and dysfunction, have been reported in the childhood cancer and ALL populations [40, 41] which parents may not always be aware of. Assessment of sleep/rest fatigue may be enhanced by the addition of an objective tool, such as actigraphy, alongside subjective questionnaire-based measures. As there is evidence that sleep quality in childhood cancer survivors is associated with fatigue, quality of life, and neurocognitive function [39, 42], further consideration of interventions which improve sleep and emphasise sleep hygiene may have benefits which are far reaching.

It was revealed that cognitive fatigue may be of particular concern to this population, given that at 27-months post-treatment this domain was significantly elevated according to both informants. Cognitive fatigue is particularly problematic in childhood cancer survivors [43] and has recently been reported to increase in patients treated for childhood ALL between active treatment and at one-year post-treatment follow up [31]. While physical activity interventions have been found to have a positive effect on fatigue in the childhood cancer population [44], one study found their exercise intervention improved general fatigue, but not cognitive fatigue [45]. Pediatric survivors of ALL attempting to meet educational demands, may therefore benefit from interventions which target the reduction and management of cognitive fatigue, and assist in coping with cognitive load.

A strength of this study is the longitudinal research design which maps the trajectory of symptoms, in contrast to the largely cross-sectional studies that exist to date. Furthermore, this study utilised both proxy- and self-reports of symptoms and included a matched comparison group with which to accurately contrast fatigue. The moderately small sample size may have been a limitation, as well as the relatively young mean age of the child sample. Consistent with papers by Varni et al., [22, 26] we found that 5–7 year olds were somewhat less reliable than adolescents in reporting on their fatigue. This possibly impacted the lack of self-reported group differences, given the unexpected variation between time points seen in the child comparison group, but not reflected in the respective parent reports. However, they still demonstrated good reliability (0.75 − 0.88), highlighting the value of measuring fatigue in this younger cohort. Additionally, there were a number of participants (*n* = 19) who were 4 years old at the first study timepoint who completed the PedsQL-MFS which has been validated for children 5 years and older. While these children were considered competent to complete the measure and were supported by the researcher to ensure they understood the survey questions, it would be valuable for future studies to determine the validity of the PedsQL-MFS for children aged 4 years given that this represents an age of peak incidence for leukemia.

## Conclusions

Children treated with chemotherapy only for ALL are at risk of experiencing fatigue over the 2-years following treatment completion according to parent reports, compared to both healthy children and normative data. Significant self-reported group differences over this period were present for general fatigue, but not total fatigue, however, patients treated for ALL went on to report significantly more total, sleep/rest, and cognitive fatigue at Time 3. Furthermore, both groups of children self-reported significantly more fatigue than the commonly utilised population normative data [26]. At 27-months post-treatment, parents and children from the patient group concur that total fatigue is significantly elevated, indicating it to be a period of increased risk for survivors who likely require appropriate screening and healthcare support. At this time, symptoms of cognitive and sleep/rest fatigue appear to be most persistent, and survivors will benefit from interventions which target these domains.

### Electronic supplementary material

Below is the link to the electronic supplementary material.

Supplementary Material 1

## Data Availability

The data that support the findings of this study are available from the corresponding author upon reasonable request. The data are not publicly available due to privacy or ethical restrictions.

## Abbreviations

ALL: Acute lymphoblastic leukemia
ITR-3: Intensity of Treatment Rating Scale
MCH: Monash Children’s Hospital
MTX: Methotrexate
RCH: Royal Children’s Hospital
PedsQL-MFS: Pediatric Quality of Life–Multidimensional Fatigue Scale
