# Supplementary material for: Fatigue in children who have recently completed treatment for acute lymphoblastic leukemia: a longitudinal study
Source: Health Qual Life Outcomes. 2024 Mar 22;22:27. doi: 10.1186/s12955-024-02241-2 (PMC10960388; doi:10.1186/s12955-024-02241-2)
Supplement: Supplementary file 1 — Supplementary Material 1 [file 12955_2024_2241_MOESM1_ESM.docx]

**Supplementary Materials**

**TABLE S1** Number of participants completing each of the age categories for the PEDSQL-MFS at the three timepoints.

|  | Time 1 | | Time 2 | | Time 3 | |
| --- | --- | --- | --- | --- | --- | --- |
| N | Patient | Comparison | Patient | Comparison | Patient | Comparison |
| Total | 77 | 52 | 75 | 45 | 68 | 43 |
| < 5 years | 11 | 8 | 0 | 0 | 0 | 0 |
| 5-7 years | 33 | 19 | 37 | 19 | 23 | 13 |
| 8-12 years | 25 | 18 | 29 | 19 | 34 | 22 |
| 13-18 years | 8 | 7 | 9 | 7 | 11 | 8 |

**TABLE S2** Results of single sample t-tests comparing fatigue scores to normative data.

|  | Patient  M (SD) | Comparison  M (SD) | Population Norm  M (SD) | Patient group compared to norm  *p* | Comparison group compared to norm  *p* |
| --- | --- | --- | --- | --- | --- |
| Child Report |  |  |  |  |  |
| *Time 1* |  |  |  |  |  |
| Total fatigue | 67.05 (19.01) | 71.47 (14.91) | 80.49 (13.33) | <.001 | <.001 |
| General fatigue | 71.49 (22.52) | 77.64 (16.59) | 85.34 (14.95) | <.001 | .002 |
| Sleep/rest fatigue | 65.30 (20.20) | 67.87 (16.92) | 75.00 (18.76) | <.001 | .004 |
| Cognitive fatigue | 64.36 (25.78) | 68.91 (21.79) | 81.14 (17.43) | <.001 | <.001 |
| *Time 2* |  |  |  |  |  |
| Total fatigue | 63.49 (17.06) | 67.31 (13.71) | 80.49 (13.33) | <.001 | <.001 |
| General fatigue | 67.87 (20.07) | 72.97 (12.71) | 85.34 (14.95) | <.001 | <.001 |
| Sleep/rest fatigue | 61.30 (20.30) | 63.76 (19.87) | 75.00 (18.76) | <.001 | .001 |
| Cognitive fatigue | 61.30 (22.75) | 65.50 (20.56) | 81.14 (17.43) | <.001 | <.001 |
| *Time 3* |  |  |  |  |  |
| Total fatigue | 63.02 (20.03) | 71.90 (13.57) | 80.49 (13.33) | <.001 | <.001 |
| General fatigue | 70.09 (20.30) | 75.68 (13.70) | 85.34 (14.95) | <.001 | <.001 |
| Sleep/rest fatigue | 59.76 (22.78) | 68.60 (17.89) | 75.00 (18.76) | <.001 | .024 |
| Cognitive fatigue | 59.51 (27.47) | 71.41 (17.92) | 81.14 (17.43) | <.001 | .001 |
| **Parent report** |  |  |  |  |  |
| *Time 1* |  |  |  |  |  |
| Total fatigue | 66.01 (17.69) | 79.90 (10.66) | 89.63 (11.38) | <.001 | <.001 |
| General fatigue | 62.77 (19.14) | 79.82 (12.89) | 89.30 (13.33) | <.001 | <.001 |
| Sleep/rest fatigue | 71.33 (17.56) | 83.66 (12.10) | 88.86 (14.72) | <.001 | .004 |
| Cognitive fatigue | 63.93 (23.04) | 76.23 (14.15) | 90.72 (15.15) | <.001 | <.001 |
| *Time 2* |  |  |  |  |  |
| Total fatigue | 71.80 (14.77) | 80.75 (13.26) | 89.63 (11.38) | <.001 | <.001 |
| General fatigue | 70.72 (16.21) | 79.07 (14.74) | 89.30 (13.33) | <.001 | <.001 |
| Sleep/rest fatigue | 77.00 (16.88) | 83.33 (12.21) | 88.86 (14.72) | <.001 | .006 |
| Cognitive fatigue | 67.69 (19.08) | 79.86 (17.77) | 90.72 (15.15) | <.001 | <.001 |
| *Time 3* |  |  |  |  |  |
| Total fatigue | 70.32 (16.57) | 81.98 (11.74) | 89.63 (11.38) | <.001 | <.001 |
| General fatigue | 68.63 (18.23) | 82.01 (11.95) | 89.30 (13.33) | <.001 | <.001 |
| Sleep/rest fatigue | 75.06 (18.16) | 82.22 (13.14) | 88.86 (14.72) | <.001 | .002 |
| Cognitive fatigue | 67.28 (21.39) | 81.71 (15.64) | 90.72 (15.15) | <.001 | .001 |

**
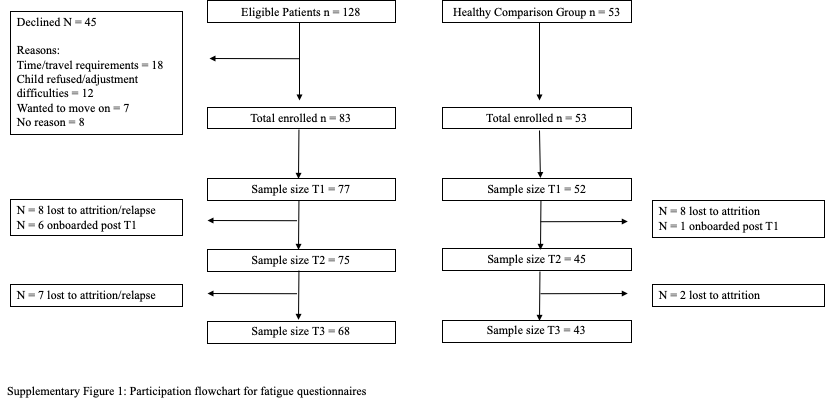
**

**FIGURE S3** Flowchart of recruitment and participation

**TABLE S4** Completion patterns

| Completion patterns, n (%) | Patients | | Comparisons | |
| --- | --- | --- | --- | --- |
| All timepoints | 63 | (75.9) | 41 | (77.4) |
| T1 & T2 | 6 | (7.2) | 2 | (3.8) |
| T1 & T3 | 0 | (0.0) | 1 | (1.9) |
| T2 & T3 | 5 | (6.0) | 1 | (1.9) |
| T1 only | 8 | (9.6) | 8 | (15.1) |
| T2 only | 1 | (1.2) | 0 | (0.0) |
| T3 only | 0 | (0.0) | 0 | (0.0) |

**TABLE S5** Child reported fatigue scores by age.

|  | Patient  M (SD) | | | Comparison  M (SD) | | |
| --- | --- | --- | --- | --- | --- | --- |
|  | *Time 1* | *Time 2* | *Time 3* | *Time 1* | *Time 2* | *Time 3* |
| Total fatigue |  |  |  |  |  |  |
| 5-7 years | 66.41 (19.73) | 62.01 (15.53) | 58.11 (21.58) | 71.19 (15.70) | 61.11 (14.45) | 67.31 (17.57) |
| 8+ years | 67.89 (18.29) | 65.01 (17.69) | 65.34 (18.91) | 71.78 (14.34) | 72.28 (11.51) | 73.89 (11.20) |
| General fatigue | |  |  |  |  |  |
| 5-7 years | 70.93 (24.15) | 65.32 (21.83) | 66.33 (24.35) | 78.40 (19.37) | 69.44 (13.71) | 75.00 (17.01) |
| 8+ years | 72.22 (20.54) | 70.49 (18.02) | 71.61 (17.80) | 76.83 (13.29) | 75.80 (11.43) | 75.97 (12.32) |
| Sleep/rest fatigue | |  |  |  |  |  |
| 5-7 years | 64.53 (23.15) | 62.16 (19.20) | 54.67 (22.45) | 69.14 (18.75) | 59.72 (27.00) | 64.10 (23.17) |
| 8+ years | 66.23 (15.84) | 64.12 (21.90) | 62.50 (22.51) | 66.50 (14.95) | 73.40 (17.52) | 70.56 (15.12) |
| Cognitive fatigue | |  |  |  |  |  |
| 5-7 years | 63.76 (26.59) | 58.56 (23.53) | 53.33 (32.98) | 66.05 (23.22) | 54.17 (19.01) | 62.82 (20.02) |
| 8+ years | 65.15 (25.06) | 64.12 (21.90) | 61.92 (24.46) | 72.00 (20.15) | 73.40 (17.52) | 75.14 (15.88) |
